# The association of human endogenous retrovirus-H long terminal repeat-associating protein 2 (HHLA2) expression with gastric cancer prognosis

**DOI:** 10.18632/oncotarget.25179

**Published:** 2018-04-24

**Authors:** Masataka Shimonosono, Takaaki Arigami, Shigehiro Yanagita, Daisuke Matsushita, Yasuto Uchikado, Yuko Kijima, Hiroshi Kurahara, Yoshiaki Kita, Shinichiro Mori, Ken Sasaki, Itaru Omoto, Kosei Maemura, Yoshikazu Uenosono, Sumiya Ishigami, Shoji Natsugoe

**Affiliations:** ^1^ Department of Digestive Surgery, Breast and Thyroid Surgery, Kagoshima University Graduate School of Medical and Dental Sciences, Kagoshima, Japan; ^2^ Department of Onco-Biological Surgery, Kagoshima University Graduate School of Medical and Dental Sciences, Kagoshima, Japan

**Keywords:** human endogenous retrovirus-H long terminal repeat-associating protein 2, quantitative reverse transcription-polymerase chain reaction, peripheral blood, prognostic marker, gastric cancer

## Abstract

Currently, immune checkpoint blockade against members of the B7/CD28 family is being used as a new molecular-targeted therapy, in patients with unresectable advanced or recurrent gastric cancer. Although human endogenous retrovirus-H long terminal repeat-associating protein 2 (HHLA2) is a novel molecule of the B7/CD28 family, the clinical impact of its expression remains uncertain in gastric cancer. Consequently, we examined HHLA2 expression in blood specimens from patients with gastric cancer, and investigated the relationship between its expression and clinicopathological factors to assess its potential power as a prognostic blood predictor. Untreated peripheral blood specimens were obtained from 111 patients with gastric cancer and 20 healthy volunteers. HHLA2 mRNA expression levels were determined using quantitative RT-PCR assay. Blood specimens obtained from patients with gastric cancer had significantly lower copies of HHLA2 mRNA than those obtained from healthy volunteers (*P* < 0.0001). Furthermore, HHLA2 expression was significantly correlated with the depth of tumor invasion (*P* = 0.0331), distant metastasis (*P* < 0.0001), and stage of disease (*P* = 0.0032). The 5-year survival rate was significantly higher in patients with high HHLA2 expression compared with the patients with low expression (*P* = 0.0001). These findings demonstrate that assessment of HHLA2 expression levels in the blood could be utilized to predict tumor aggressiveness in patients with gastric cancer.

## INTRODUCTION

Gastric cancer is the fifth most frequent malignancy and one of the leading causes of cancer-specific mortality worldwide [1]. Although the mortality rate of gastric cancer has been continuously decreasing in Japan, it is still responsible for the third highest mortality rate [2]. On the other hand, the remarkable advances in chemotherapy have contributed greatly to improved prognosis in patients with unresectable advanced or recurrent gastric cancer [3, 4]. Furthermore, drugs such as trastuzumab and ramucirumab, which have molecular targets, have been clinically used in the therapeutic management of patients with unresectable advanced or recurrent gastric cancer [5, 6]. However, the 5-year survival rates of patients with American Joint Committee on Cancer stage IIIA, stage IIIB, stage IIIC, and stage IV are 58.4%, 40.8%, 20.2%, and 8.8%, respectively [7]. These findings indicate a limitation of current treatments, including surgery and chemotherapy, in the clinical strategy for advanced gastric cancer.

Recently, an immune-checkpoint inhibitor was discovered and clinical studies demonstrated its efficacy in patients with several malignancies, including gastric cancer [8]. The ATTRACTION-2 trial showed the survival benefits of nivolumab, an anti-programmed cell death protein 1 (PD-1) antibody, in patients with pre-treated advanced gastric or gastro-esophageal junction cancer [9]. This phase 3 study indicated that the median overall survival was 5.26 and 4.14 months in the nivolumab and placebo groups, respectively. PD-1 is a receptor for programmed death ligand-1 in the B7 family pathway and these molecules play an important role in the inhibition of the T-cell-mediated immune response in patients with malignancies [10]. To date, investigators have identified several immune molecules for controlling T-cell-mediated immune response toward the B7 family pathway [10, 11]. Consequently, these immune molecules are gathering attention as new therapeutic targets for promoting the development of cancer immunotherapy. Previously, we reported that B7-H3 and B7-H4, members of the B7 family, were highly expressed in resected tumor tissues and peripheral blood specimens obtained from patients with gastric cancer [12, 13]. Moreover, our studies demonstrated a positive correlation between these mRNA levels and tumor progression or prognosis. Currently, promising blood markers for predicting tumor aggressiveness are clinically limited in patients with unresectable advanced or recurrent gastric cancer. Therefore, the discovery of new immune molecules that are present in the blood may be important for the clinical management of such patients.

Human endogenous retrovirus-H long terminal repeat-associating protein 2 (HHLA2) is the most recently discovered member of the B7 family, also known as B7-H5 or B7-H7 [14, 15]. Recent studies have investigated the expression of HHLA2 in various cancer cells [16–18]. However, there have been no studies assessing the clinical significance of HHLA2 expression in blood specimens and tumor tissues obtained from patients with gastric cancer.

The purpose of the current study was to examine the expression of HHLA2 in blood specimens and to compare the status of its expression between blood and primary tumor specimens obtained from patients with gastric cancer. Furthermore, we investigated the relationship between HHLA2 expression and tumor behavior to assess its clinical utility as a blood prognostic predictor.

## RESULTS

### Quantitative reverse transcription-polymerase chain reaction (qRT-PCR) analysis of HHLA2 mRNA levels in blood specimens

**Figure 1:**
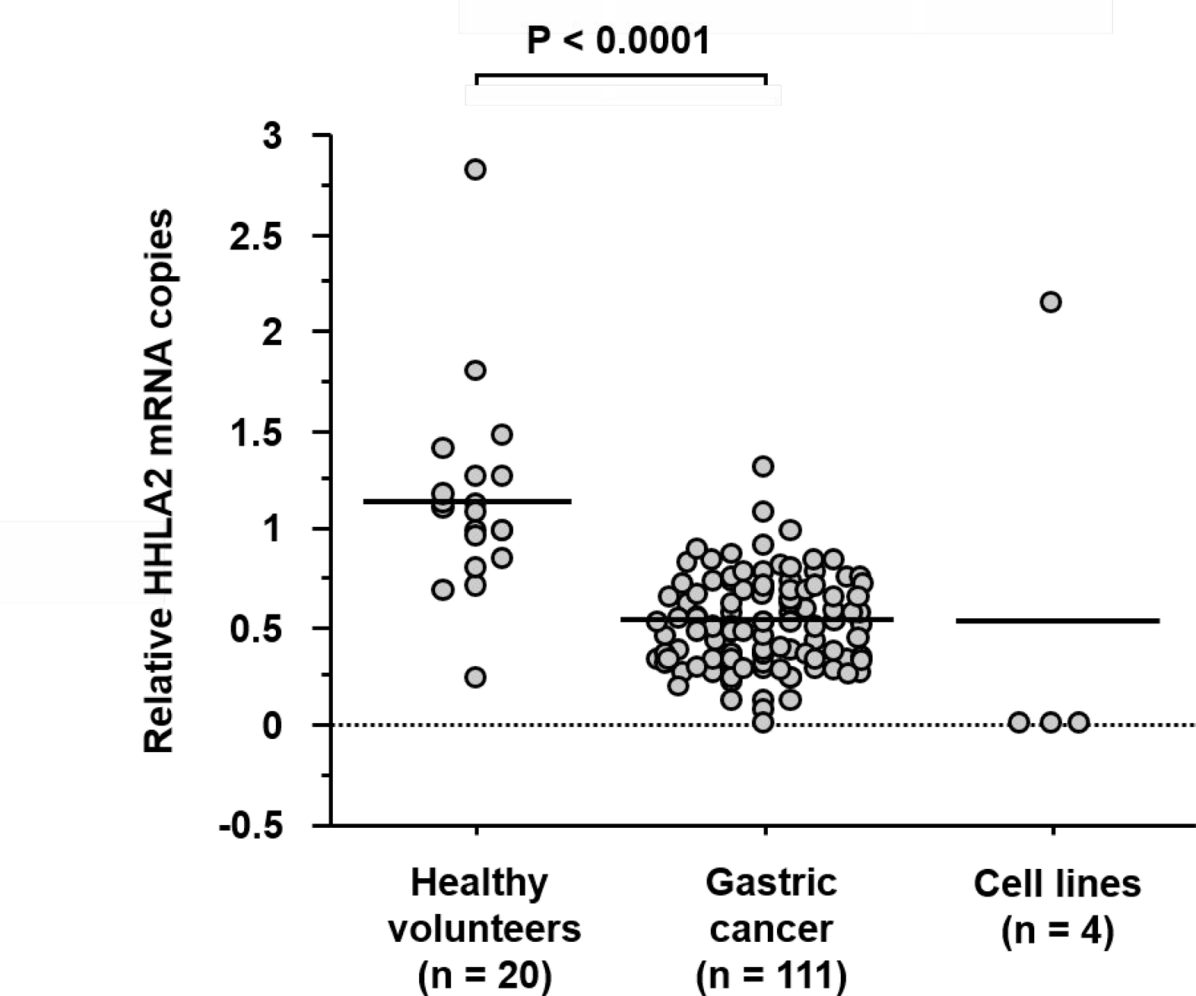
qRT-PCR analysis of HHLA2 mRNA expression levels in blood specimens GAPDH was used for normalization and the relative expression levels were analyzed using the 2−ΔΔCT method. HHLA2 mRNA levels were significantly lower in blood specimens obtained from patients with gastric cancer than in PBMC specimens obtained from healthy volunteers (*P* < 0.0001).

We evaluated HHLA2 mRNA levels using the qRT-PCR assay in four gastric cancer cell lines, 111 blood specimens obtained from patients with gastric cancer, and 20 peripheral blood mononuclear cell (PBMC) specimens obtained from healthy volunteers. Of the four gastric cancer cell lines, only MKN-45 showed expression of HHLA2 mRNA. In the blood specimens obtained from patients with gastric cancer, the relative number of HHLA2 mRNA copies ranged from 0.0214 to 1.30. In the PBMC specimens obtained from healthy volunteers, the relative number of HHLA2 mRNA copies ranged from 0.25 to 2.81. The mean value of the relative HHLA2 mRNA copies (±SD) in the blood specimens and PBMC specimens was 0.515 ± 0.228 and 1.14 ± 0.509, respectively (Figure 1). HHLA2 mRNA levels were significantly lower in blood specimens obtained from patients than in PBMC specimens obtained from healthy volunteers (*P* < 0.0001).

### HHLA2 expression and clinicopathological factors

Based on the median value of the relative HHLA2 mRNA copies, 111 patients with gastric cancer were divided into a high-expression group (*n* = 55) and a low-expression group (*n* = 56) to investigate the relationship between expression of HHLA2 and clinicopathological factors. HHLA2 expression showed a significant negative correlation with the depth of tumor invasion, distant metastasis, and tumor-node-metastasis (TNM) stage (*P* = 0.0331, *P* < 0.0001, and *P* = 0.0032, respectively) (Table 1).

**Table 1:** HHLA2 expression and clinicopathological factors

Factor	HHLA2 expression (%)		*P*-value
	Low (*n* = 55)	High (*n* = 56)	
Gender			
Male	36 (65.5)	36 (64.3)	0.8974
Female	19 (34.5)	20 (35.7)	
Age (year)			
<70	27 (49.1)	40 (71.4)	0.0161
≥70	28 (50.9)	16 (28.6)	
Tumor location			
Upper	17 (30.9)	16 (28.6)	0.1915
Middle	17 (30.9)	26 (46.4)	
Lower	21 (38.2)	14 (25.0)	
Histological type			
Differentiated	25 (45.5)	19 (33.9)	0.2145
Undifferentiated	30 (54.5)	37 (66.1)	
Depth of tumor invasion			
T1	18 (32.7)	27 (48.2)	0.0331
T2	3 (5.5)	3 (5.4)	
T3	5 (9.1)	11 (19.6)	
T4	29 (52.7)	15 (26.8)	
Lymph node metastasis			
Negative	30 (54.5)	34 (60.7)	0.5108
Positive	25 (45.5)	22 (39.3)	
Distant metastasis			
Negative	26 (47.3)	47 (83.9)	<0.0001
Positive	29 (52.7)	9 (16.1)	
Stage			
I–II	23 (41.8)	39 (69.6)	0.0032
III–IV	32 (58.2)	17 (30.4)	
Serum CEA levels (>5 ng/mL)			
Negative	40 (72.7)	48 (85.7)	0.0914
Positive	15 (27.3)	8 (14.3)	
Serum CA19-9 levels (>37 U/mL)			
Negative	45 (81.8)	50 (89.3)	0.2627
Positive	10 (18.2)	6 (10.7)	
Lymphatic invasion (*n* = 73)			
Negative	18 (66.7)	30 (65.2)	0.8998
Positive	9 (33.3)	16 (34.8)	
Venous invasion (*n* = 73)			
Negative	20 (74.1)	30 (65.2)	0.4316
Positive	7 (25.9)	16 (34.8)	

CEA: carcinoembryonic antigen; CA 19-9: carbohydrate antigen 19-9; T1: invasion of lamina propria or submucosa; T2: invasion of muscularis propria; T3: invasion of subserosa; T4: penetration of serosa or invasion of adjacent structures.

### HHLA2 expression and prognosis

**Figure 2:**
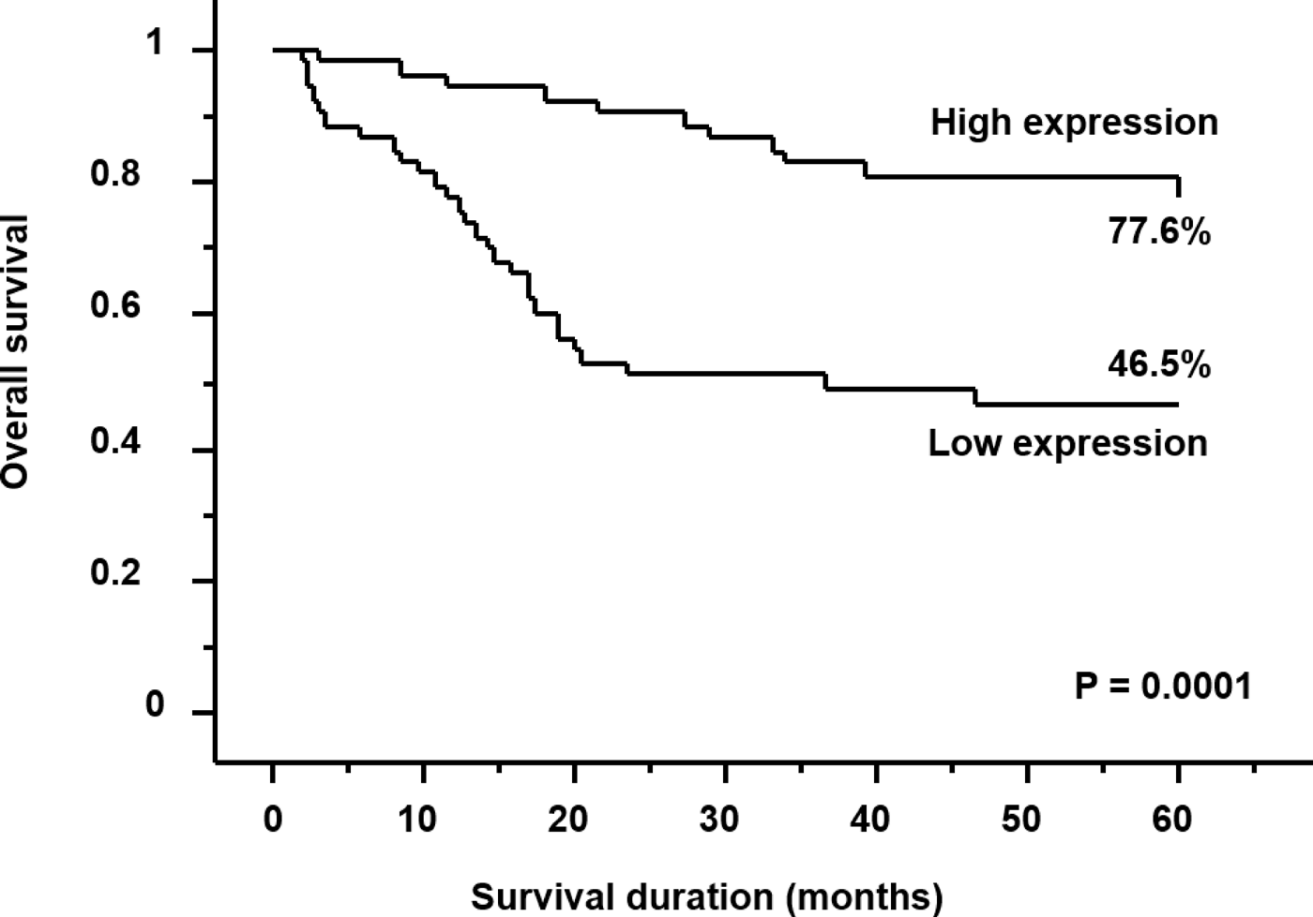
Kaplan–Meier survival curves for patients with gastric cancer Patients with high HHLA2 expression had a significantly better prognosis than those with low HHLA2 expression (*P* = 0.0001).

The 5-year survival rate was significantly higher in the high-expression group compared with that in the low-expression group (77.6% vs. 46.5%, respectively; *P* = 0.0001) (Figure 2). Univariate analysis showed that the depth of tumor invasion, lymph node metastasis, distant metastasis, level of serum carcinoembryonic antigen (CEA), level of serum carbohydrate antigen (CA) 19-9, and HHLA2 expression were significantly related to overall survival (*P* < 0.0001, *P* = 0.0001, *P* < 0.0001, *P* < 0.0001, *P* < 0.0001, and *P* = 0.0004, respectively) (Table 2). Multivariate analysis indicated that distant metastasis and the level of serum CEA were independent prognostic factors (*P* = 0.0004 and *P* = 0.0162, respectively) (Table 2). Thus, expression of HHLA2 was not an independent prognostic factor according to the multivariate analysis (*P* = 0.6149).

**Table 2:** Univariate and multivariate analyses of survival

Factor	Univariate analysis			Multivariate analysis		
	Hazard ratio	95% CI	*P*-value	Hazard ratio	95% CI	*P*-value
Depth of tumor invasion T1–2/T3–4	21.86	5.26–90.9	<0.0001	1.13	0.1–12.79	0.9224
Lymph node metastasis Negative/Positive	3.86	1.95–7.63	0.0001	1.18	0.56–2.46	0.6679
Distant metastasis Negative/Positive	65.52	19.39–221.4	<0.0001	43.35	5.477–343.04	0.0004
Serum CEA levels (>5 ng/mL) Negative/Positive	6.21	3.26–11.84	<0.0001	2.34	1.17–4.66	0.0162
Serum CA 19-9 levels (>37 U/mL) Negative/Positive	6.41	3.21–12.81	<0.0001	1.21	0.59–2.45	0.6024
HHLA2 expression Low/High	0.28	0.14–0.56	0.0004	0.82	0.39–1.76	0.6149

CEA: carcinoembryonic antigen; CA 19-9: carbohydrate antigen 19-9; CI: confidence interval; T1: invasion of lamina propria or submucosa; T2: invasion of muscularis propria; T3: invasion of subserosa; T4: penetration of serosa or invasion of adjacent structures.

### Immunohistochemical analysis of HHLA2 protein expression in primary tumor specimens

**Figure 3:**
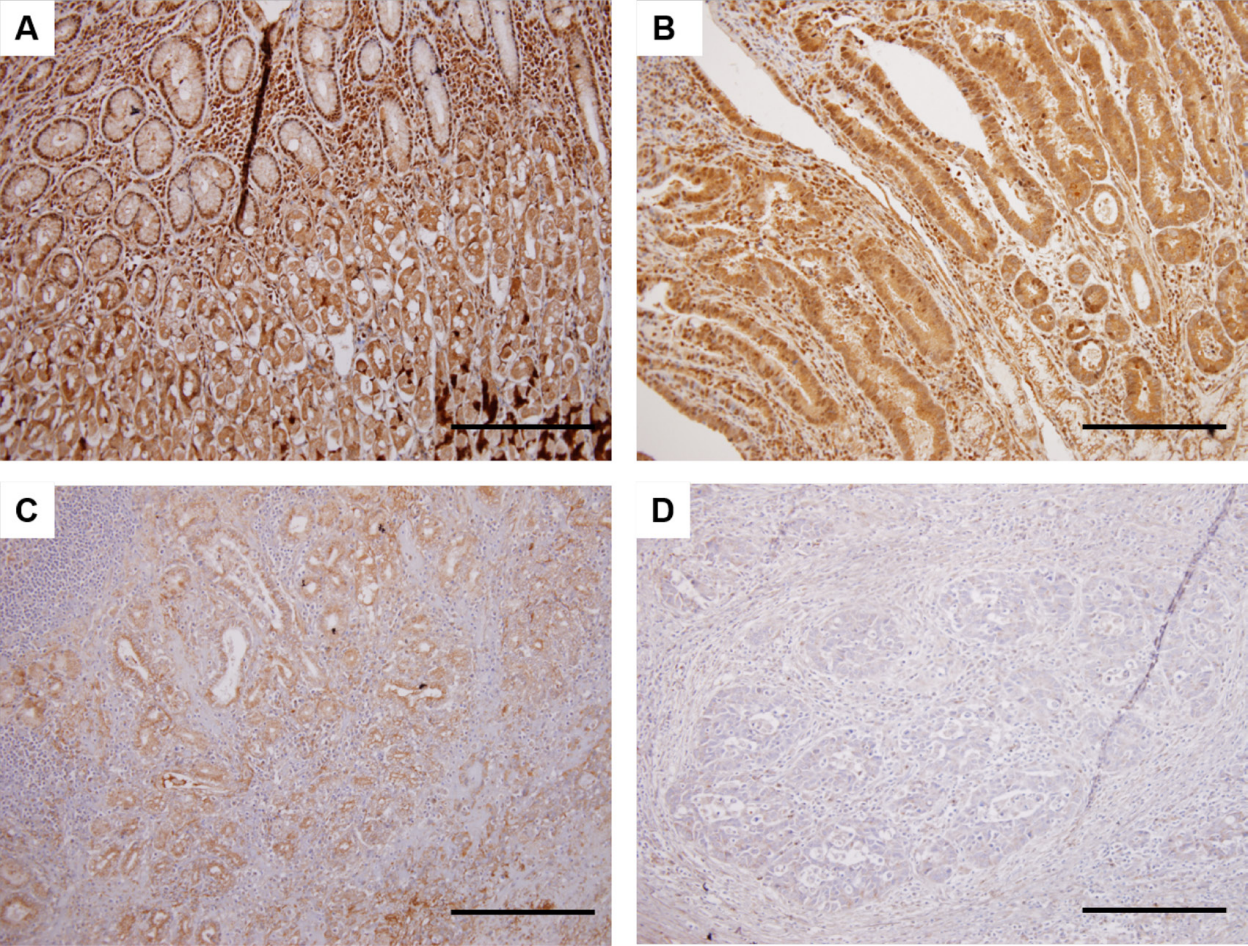
Immunohistochemical analysis for HHLA2 protein expression in primary tumor specimens On the basis of staining intensity, the status of HHLA2 expression was classified into the following three groups: high, intermediate, and low immunoreactions. (**A**) HHLA2 protein expression in normal epithelial cells. (**B**) Tumor cells with high expression of HHLA2. (**C**) Tumor cells with intermediate expression of HHLA2. (**D**) Tumor cells with low expression of HHLA2. Scale bars indicate 200 μm (Original magnification ×200).

**Figure 4:**
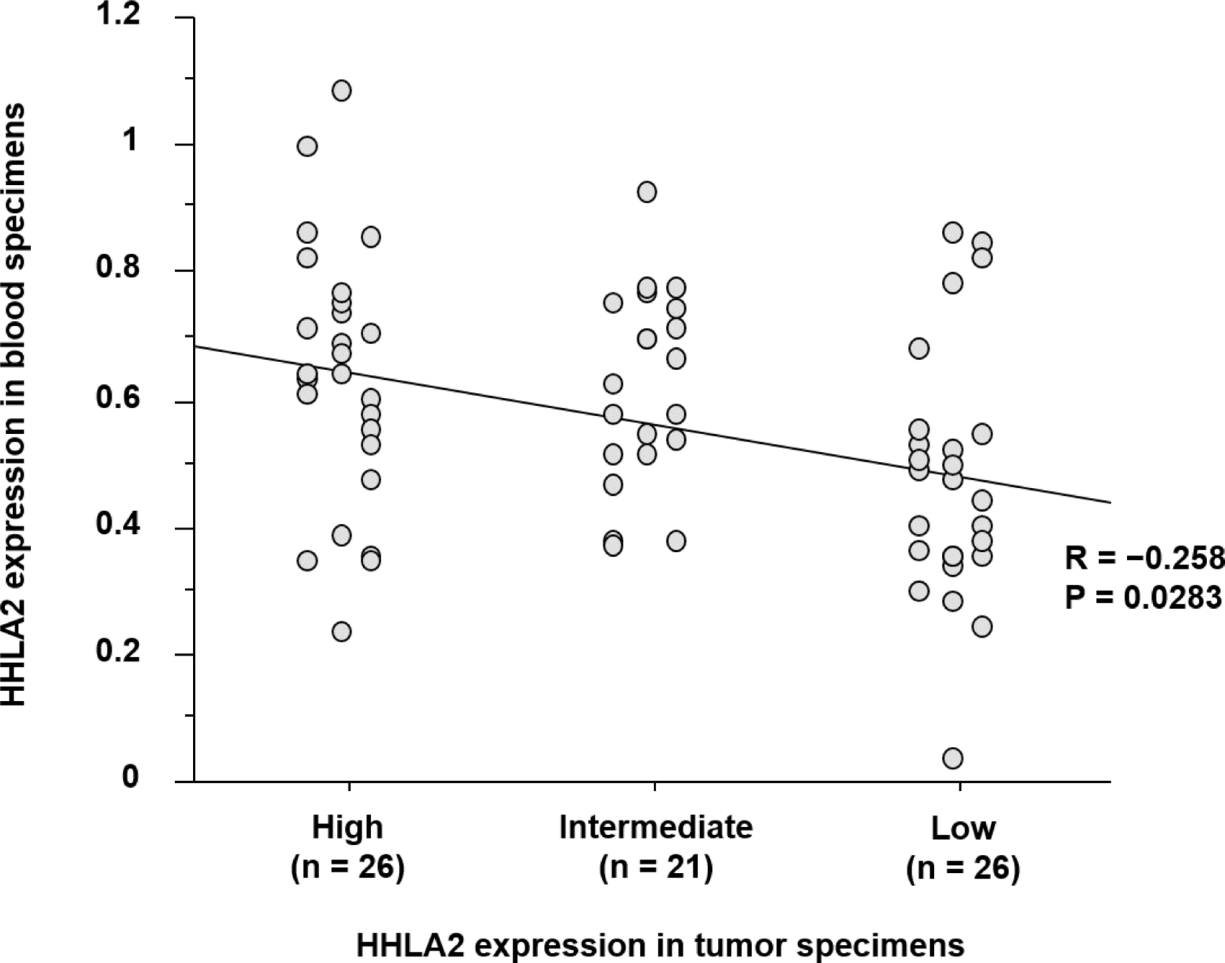
Correlation of the status of HHLA2 expression between blood specimens and primary tumor tissues A comparative analysis based on qRT-PCR and immunohistochemistry demonstrated a weak linear relationship in HHLA2 expression between blood specimens and primary tumor tissues (*r* = −0.258, *P* = 0.0283).

HHLA2 protein expression was assessed by immunohistochemistry of 73 tumor tissues resected from patients with gastric cancer. Immunohistochemical analysis demonstrated that HHLA2 was mainly expressed in the membrane and/or cytoplasm of normal epithelial cells and tumor cells (Figure 3). Normal epithelial cells showed high HHLA2 expression (Figure 3A). According to the immunoreactivity of HHLA2, patient samples were classified as tissues with high (*n* = 26; 35.6%), tissues with intermediate (*n* = 21; 28.8%), and tissues with low (*n* = 26; 35.6%) expressions (Figure 3B–3D, respectively). The mean values of the relative HHLA2 mRNA copies (±SD) in the high, intermediate, and low-expression groups were 0.625 ± 0.204, 0.605 ± 0.152, and 0.463 ± 0.197, respectively. Spearman’s rank test showed a weak linear relationship in HHLA2 expression between blood specimens and primary tumor tissues (*r* = −0.258, *P* = 0.0283) (Figure 4).

## DISCUSSION

HHLA2 was identified in 1999 as a new molecule containing immunoglobulin-like domains [19]. Recently, it was revealed that HHLA2 has a 10%‒18% amino acid identity and 23%–33% similarity to other B7 family members, and it was classified as a new B7/CD28 family member [20]. HHLA2 protein is constitutively expressed on the surface of human monocytes or macrophages, and it is not expressed on immature dendritic cells and resting T- or B-cells [14, 20]. Furthermore, Janakiram *et al.* showed that HHLA2 is expressed in 20%‒70% of human cancers such as breast, lung, thyroid, melanoma, pancreas, ovary, liver, bladder, colon, prostate, kidney, and esophagus [16]. Furthermore, Koirala *et al.* reported that HHLA2 was expressed in 68% of patients with osteosarcoma [17]. On the contrary, based on immunohistochemical staining, Byers *et al.* reported that HHLA2 expression was decreased or absent in pancreatic adenocarcinoma tissue, although highly expressed in normal ductal epithelium or intraductal papillary mucinous neoplasms [18]. In the current study, we investigated HHLA2 protein expression in resected tumor tissues obtained from patients with gastric cancer, including normal epithelium of the stomach, using immunohistochemistry. Surprisingly, normal epithelial cells showed high expression of HHLA2 compared with tumor cells. These findings indicate a close similarity in the pattern of HHLA2 expression between gastric and pancreatic cancers.

In this study, we focused on the clinical significance of HHLA2 expression in blood specimens obtained from patients with gastric cancer. To our knowledge, no study has examined the clinical impact of its expression in blood specimens. Currently, blood specimens have been attracting attention as an important internal product for repeatable liquid biopsy in the clinical management of patients with various malignancies. Therefore, we assessed HHLA2 mRNA levels using qRT-PCR assay in blood specimens obtained from patients with gastric cancer and PBMC specimens obtained from healthy volunteers. Interestingly, HHLA2 mRNA levels were significantly lower in the blood specimens compared with those in the PBMC specimens (*P* < 0.0001). Furthermore, we confirmed the absence of HHLA2 expression in the majority of gastric cancer cell lines that were examined. Consequently, expression of HHLA2 in blood specimens may reflect the systemic immune status relating to circulating tumor cells and antigen presenting cells (APCs). These results suggest that the assessment of HHLA2 expression in blood specimens could be utilized to differentiate patients with gastric cancer from healthy volunteers.

In addition, we examined the relationship between HHLA2 mRNA levels and clinicopathological factors. HHLA2 expression was inversely related with the depth of tumor invasion, distant metastasis, and stage of disease. Accordingly, HHLA2 expression was associated with less aggressive tumor behavior in patients with gastric cancer. However, patients with high expression of HHLA2 have a higher risk of lymph node metastasis and advanced breast cancer. In patients with osteosarcoma, high HHLA2 expression was associated with a significantly worse prognosis [16, 17]. These findings may indicate a different molecular mechanism of HHLA2 in each tumor. According to recent studies, HHLA2 has two receptors on T-cells [21], namely the transmembrane and immunoglobulin domain-containing 2 (TMIGD2) receptor and another receptor, which remains unknown [14, 16]. Consequently, HHLA2 has costimulatory and coinhibitory effects against the T-cell-mediated immune response via TMIGD2 and the unknown receptor, respectively [21]. HHLA2 on APCs costimulates naïve T-cell proliferation and cytokine production through TMIGD2 via an AKT-dependent signaling cascade [14]. As activated T-cells lose TMIGD2 expression, the second receptor for HHLA2 exerts a coinhibitory effect on these activated T-cells [20, 22]. Accordingly, HHLA2 may exert a costimulatory effect against the T cell-mediated immune response via TMIGD2 in patients with gastric cancer. These findings may suggest that gastric tumor cells acquire immune evasion by the reduction of own HHLA2 expression and that these cells have aggressive behavior.

In the current study, the 5-year survival rate was 77.6% and 46.5% in the high-expression group and in the low-expression group, respectively (*P* = 0.0001). Multivariate analysis demonstrated that the expression of HHLA2 was not an independent prognostic factor (*P* = 0.6149). This may be result of the limitations of the current study. This preliminary study was conducted in a single institution and was based on a retrospective analysis of a small population (*n* = 111). Moreover, the median follow-up period was only 39.2 months. Validation studies with larger samples sizes are warranted to confirm our hypothesis. However, the univariate analysis showed HHLA2 expression as an independent prognostic factor (*P* = 0.0004). Thus, HHLA2 may be a promising blood marker for predicting prognosis after treatment in patients with gastric cancer.

Although the immunological role of the HHLA2 signaling pathway remains unclear, a new technique that allows gastric tumor cells to express HHLA2 may enable a targeted immunotherapy that controls its signaling pathway in patients with unresectable advanced gastric cancer. In this study, we compared differences in HHLA2 expression between blood specimens and primary tumor tissues in each patient. Immunohistochemical analysis demonstrated that HHLA2 mRNA levels in blood specimens were weakly correlated with HHLA2 protein expression in primary tumor tissues (*r* = −0.258, *P* = 0.0283). Supposing the assessment of HHLA2 expression in the primary tumor site is a tool for predicting tumor response against HHLA2-targeted immunotherapy, a blood biopsy to determine HHLA2 expression could be utilized to monitor sequential therapeutic effects in patients with advanced gastric cancer.

In conclusion, the findings of this study demonstrate that HHLA2 expression is related to tumor progression and prognosis in patients with gastric cancer. Therefore, the HHLA2 molecule may be a potential biomarker for predicting malignant behavior in patients with gastric cancer.

## MATERIALS AND METHODS

### Gastric cancer cell lines

Four gastric cancer cell lines, namely MKN-7, MKN-45, MKN-74, and KATO-III (Japanese Physical and Chemical Institute, Tokyo, Japan), were used in this study. Cell lines were cultured in RPMI 1640 (Nissui Pharmaceutical Co. Ltd., Tokyo, Japan) supplemented with 10% fetal calf serum (Mitsubishi Kasei, Tokyo, Japan), 100 units ⁄mL penicillin and 100 units⁄mL streptomycin, and incubated at 37° C in a humidified atmosphere containing 5% CO_2_, as previously described [12, 13].

### Patients

Whole blood specimens were obtained from 111 patients (72 males and 39 females; age range: 37–86 years; average age: 66 years) with gastric cancer at Kagoshima University Hospital (Kagoshima, Japan) between 2010 and 2015. All blood specimens were collected prior to treatment. Seventy-three patients underwent curative gastrectomy with lymphadenectomy, and the remaining 38 patients with unresectable advanced or recurrent gastric cancer received chemotherapy and/or radiotherapy or the best possible supportive care. PBMC specimens from 20 healthy volunteers served as the control group. Paraffin-embedded archival tissue specimens of resected primary gastric tumors obtained from 73 patients were used for immunohistochemistry. Patients were grouped and staged based on the Union for International Cancer Control criteria of TNM classification for gastric carcinoma [23]. Moreover, lymphatic and venous invasions were defined on the basis of the criteria of Japanese classification for gastric carcinoma (3rd English edition) [24]. The median follow-up period was 39.2 months (range: 2–60 months).

The current study was approved by the Ethics Committee of Kagoshima University (approval number: 1676). Written informed consent and approval were obtained from all patients.

### Enzyme immunoassay for the determination of serum CEA and CA 19-9

Serum concentrations of CEA and CA19-9 were examined using a commercial enzyme immunoassay kit (Abbott Co., Ltd., Tokyo, Japan). A serum CEA level of >5.0 ng/mL and a serum CA 19-9 level of >37 U/mL were defined as positive according to the manufacturer’s instructions.

### Blood processing and RNA extraction

Blood specimens (5 mL) were collected from all the patients in tubes containing sodium citrate. The samples were processed within 1 h after collection. Next, PBMC specimens were separated using a lymphocyte separation medium (MP Biomedicals, Santa Ana, CA, USA) according to the manufacturer’s protocol. All processed blood specimens and harvested cell lines were mixed with Isogen (Nippon Gene, Toyama, Japan) and immediately cryopreserved at −80° C until RNA extraction. Total RNA was isolated and purified using phenol-chloroform extraction, as previously described [12, 13]. The quality of RNA was assessed using a NanoDrop Lite UV-Vis Spectrophotometer (Thermo Fisher Scientific, Wilmington, DE, USA).

### qRT-PCR assay

Complementary DNA (cDNA) was synthesized using a High-Capacity cDNA Reverse Transcription Kit (Applied Biosystems, Waltham, MA, USA). Gene-specific PCR products were continuously assessed using a Step One Plus Real-Time PCR System (Applied Biosystems) according to the manufacturer’s protocol. TaqMan^®^ probes and primers for HHLA2 (P/N: Hs00978112_m1) and glyceraldehyde-3-phosphatase dehydrogenase (GAPDH) (P/N: Hs99999905_m1) were obtained from Applied Biosystems. GAPDH was used for normalization. The relative expression levels were analyzed using the 2−ΔΔCT method [25]. Each assay was performed in duplicate, including negative control reactions that lacked cDNA.

### Immunohistochemistry

Tumor specimens were fixed with 10% formaldehyde in phosphate-buffered saline (PBS), embedded in paraffin, and sectioned into 4 μm-thick slices. Immunohistochemical staining was performed using the avidin-biotin-peroxidase complex method (Vectastatin Elite ABC kit; Vector, Burlingame, CA, USA) according to the manufacturer’s instructions. The sections were deparaffinized in xylene and dehydrated in ethanol. Next, the sections were autoclaved in citrate buffer (0.01 mol/L, pH 6.5) at 121° C for 10 min to reveal the antigen. After cooling, endogenous peroxidase activity was blocked by incubating sections for 10 min in 0.3% hydrogen peroxide. Non-specific binding was blocked at room temperature for 20 min with normal goat serum. Sections were incubated at 4° C overnight with anti-HHLA2 rabbit polyclonal antibody (ab214327; Abcam, Cambridge, UK) diluted 1:200 in antibody diluent (Dako REAL antibody diluent, Santa Clara, CA, USA). After rinsing with PBS for 5 min, the sections were incubated with secondary antibody for 30 min and washed once more. After washing with PBS for 5 min, the sections were incubated with avidin-biotin complex for 30 min and washed once more. Reactions were visualized using diaminobenzidine tetrahydrochloride for 5 min. All samples were lightly counterstained with hematoxylin for 20 sec.

Sections treated without primary antibody were used as a negative control and the human rectal carcinoma tissue was used as a positive control. Based on the staining intensity, the status of HHLA2 expression was divided into three groups: high, intermediate, and low [18].

### Statistical analysis

The statistical analysis for differences in HHLA2 mRNA levels between PBMC specimens from healthy volunteers and blood specimens from patients with gastric cancer was performed using the Mann‒Whitney *U*-test. Spearman’s rank test was used to compare the status of HHLA2 expression between blood specimens and primary tumor tissues. In the comparison of clinicopathological factors, group differences were analyzed using the chi-squared and Fisher’s exact tests. Overall survival curves were plotted according to the Kaplan‒Meier method and the prognostic difference was analyzed using the log-rank test. Prognostic factors were analyzed by univariate and multivariate analyses using the Cox proportional hazard regression model. All statistical analyses were performed using the Expert StatView version 5.0 software (SAS Institute, Inc., Cary, NC, USA). A *P-*value of < 0.05 was considered statistically significant.

## References

[1] Torre LA, Bray F, Siegel RL, Ferlay J, Lortet-Tieulent J, Jemal A (2015). Global cancer statistics, 2012. CA Cancer J Clin.

[2] Hori M, Matsuda T, Shibata A, Katanoda K, Sobue T, Nishimoto H, Japan Cancer Surveillance Research Group (2015). Cancer incidence and incidence rates in Japan in 2009: a study of 32 population-based cancer registries for the Monitoring of Cancer Incidence in Japan (MCIJ) project. Japanese Journal of Clinical Oncology.

[3] Koizumi W, Narahara H, Hara T, Takagane A, Akiya T, Takagi M, Miyashita K, Nishizaki T, Kobayashi O, Takiyama W, Toh Y, Nagaie T, Takagi S (2008). S-1 plus cisplatin versus S-1 alone for first-line treatment of advanced gastric cancer (SPIRITS trial): a phase III trial. Lancet Oncol.

[4] Yamada Y, Higuchi K, Nishikawa K, Gotoh M, Fuse N, Sugimoto N, Nishina T, Amagai K, Chin K, Niwa Y, Tsuji A, Imamura H, Tsuda M (2015). Phase III study comparing oxaliplatin plus S-1 with cisplatin plus S-1 in chemotherapy-naïve patients with advanced gastric cancer. Annals of Oncology.

[5] Bang YJ, Van Cutsem E, Feyereislova A, Chung HC, Shen L, Sawaki A, Lordick F, Ohtsu A, Omuro Y, Satoh T, Aprile G, Kulikov E, Hill J (2010). Trastuzumab in combination with chemotherapy versus chemotherapy alone for treatment of HER2-positive advanced gastric or gastro-oesophageal junction cancer (ToGA): a phase 3, open-label, randomised controlled trial. Lancet.

[6] Wilke H, Muro K, Van Cutsem E, Oh SC, Bodoky G, Shimada Y, Hironaka S, Sugimoto N, Lipatov O, Kim TY, Cunningham D, Rougier P, Komatsu Y (2014). Ramucirumab plus paclitaxel versus placebo plus paclitaxel in patients with previously treated advanced gastric or gastro-oesophageal junction adenocarcinoma (RAINBOW): a double-blind, randomised phase 3 trial. Lancet Oncol.

[7] Kim SG, Seo HS, Lee HH, Song KY, Park CH (2017). Comparison of the Differences in Survival Rates between the 7th and 8th Editions of the AJCC TNM Staging System for Gastric Adenocarcinoma: a Single-Institution Study of 5,507 Patients in Korea. Journal of Gastric Cancer.

[8] Gong J, Chehrazi-Raffle A, Reddi S, Salgia R (2018). Development of PD-1 and PD-L1 inhibitors as a form of cancer immunotherapy: a comprehensive review of registration trials and future considerations. Journal for Immunotherapy of Cancer.

[9] Kang YK, Boku N, Satoh T, Ryu MH, Chao Y, Kato K, Chung HC, Chen JS, Muro K, Kang WK, Yeh KH, Yoshikawa T, Oh SC (2017). Nivolumab in patients with advanced gastric or gastro-oesophageal junction cancer refractory to, or intolerant of, at least two previous chemotherapy regimens (ONO-4538-12, ATTRACTION-2): a randomised, double-blind, placebo-controlled, phase 3 trial. Lancet.

[10] Zang X, Allison JP (2007). The B7 family and cancer therapy: costimulation and coinhibition. Clinical Cancer Research.

[11] Ni L, Dong C (2017). New B7 Family Checkpoints in Human Cancers. Molecular Cancer Therapeutics.

[12] Arigami T, Uenosono Y, Hirata M, Yanagita S, Ishigami S, Natsugoe S (2011). B7-H3 expression in gastric cancer: a novel molecular blood marker for detecting circulating tumor cells. Cancer Science.

[13] Arigami T, Uenosono Y, Ishigami S, Hagihara T, Haraguchi N, Natsugoe S (2011). Clinical significance of the B7-H4 coregulatory molecule as a novel prognostic marker in gastric cancer. World Journal of Surgery.

[14] Zhu Y, Yao S, Iliopoulou BP, Han X, Augustine MM, Xu H, Phennicie RT, Flies SJ, Broadwater M, Ruff W, Taube JM, Zheng L, Luo L (2013). B7-H5 costimulates human T cells via CD28H. Nature Communications.

[15] Flajnik MF, Tlapakova T, Criscitiello MF, Krylov V, Ohta Y (2012). Evolution of the B7 family: co-evolution of B7H6 and NKp30, identification of a new B7 family member, B7H7, and of B7’s historical relationship with the MHC. Immunogenetics.

[16] Janakiram M, Chinai JM, Fineberg S, Fiser A, Montagna C, Medavarapu R, Castano E, Jeon H, Ohaegbulam KC, Zhao R, Zhao A, Almo SC, Sparano JA (2015). Expression, clinical significance, and receptor identification of the newest B7 family member HHLA2 protein. Clinical Cancer Research.

[17] Koirala P, Roth ME, Gill J, Chinai JM, Ewart MR, Piperdi S, Geller DS, Hoang BH, Fatakhova YV, Ghorpade M, Zang X, Gorlick R (2016). HHLA2, a member of the B7 family, is expressed in human osteosarcoma and is associated with metastases and worse survival. Scientific Reports.

[18] Byers JT, Paniccia A, Kaplan J, Koenig M, Kahn N, Wilson L, Chen L, Schulick RD, Edil BH, Zhu Y (2015). Expression of the Novel Costimulatory Molecule B7-H5 in Pancreatic Cancer. Annals of Surgical Oncology.

[19] Mager DL, Hunter DG, Schertzer M, Freeman JD (1999). Endogenous retroviruses provide the primary polyadenylation signal for two new human genes (HHLA2 and HHLA3). Genomics.

[20] Zhao R, Chinai JM, Buhl S, Scandiuzzi L, Ray A, Jeon H, Ohaegbulam KC, Ghosh K, Zhao A, Scharff MD, Zang X (2013). HHLA2 is a member of the B7 family and inhibits human CD4 and CD8 T-cell function. Proc Natl Acad Sci U S A.

[21] Xiao Y, Freeman GJ (2015). A New B7:CD28 Family Checkpoint Target for Cancer Immunotherapy: HHLA2. Clinical Cancer Research.

[22] Wang J, Manick B, Wu G, Hao R (2014). Biofunctions of three new B7 family members (IRM7P.486). J Immunol.

[23] Edge SB, Byrd DR, Compton CC, Fritz AG, Greene FL, Trotti A (2010). AJCC Cancer Staging Manual, 7th ed.

[24] Japanese Gastric Cancer Association (2011). Japanese classification of gastric carcinoma: 3rd English edition. Gastric Cancer.

[25] Livak KJ, Schmittgen TD (2001). Analysis of relative gene expression data using real-time quantitative PCR and the 2(-Delta Delta C(T)) Method. Methods.

